# Comparing financing models for supplementary healthcare in appendectomy: activity-based costing (fee-for-service) vs. diagnosis related group remuneration (*bundled payment*) – a systematic review and meta-analysis

**DOI:** 10.1590/acb386923

**Published:** 2023-12-04

**Authors:** André de Arimatéia de Souza Lino, Jose Arnaldo Shiomi da Cruz, Breno Cordeiro Porto, Rhuan Pimentel Nogueira, José Pinhata Otoch, Everson Luiz de Almeida Artifon

**Affiliations:** 1 Universidade de São Paulo – School of Medicine – Surgical Technique and Experimental Surgery – São Paulo (SP) – Brazil.; 2 Hospital Alemão Oswaldo Cruz – Specialized Center for Urology – São Paulo (SP) – Brazil.

**Keywords:** Remuneration, Health Services, Prospective Payment System, Fee-for-Service Plans

## Abstract

**Purpose:**

In Brazil, healthcare services traditionally follow a fee-for-service (FFS) payment system, in which each medical procedure incurs a separate charge. An alternative reimbursement with the aim of reducing costs is diagnosis related group (DRG) remuneration, in which all patient care is covered by a fixed amount. This work aimed to perform a systematic review followed by meta-analysis to assess the effectiveness of the Budled Payment for Care Improvement (BPCI) versus FFS.

**Methods:**

Our work was performed following the items of the PRISMA report. We included only observational trials, and the primary outcome assessed was the effectiveness of FFS and DRG in appendectomy considering complications. We also assessed the costs and length of hospital stay. Meta-analysis was performed with Rev Man version 5.4.

**Results:**

Out of 735 initially identified articles, six met the eligibility criteria. We demonstrated a shorter hospital stay associated with the DRG model (mean difference = 0.39; 95% confidence interval – 95%CI – 0.38–0.40; p < 0.00001; I2 = 0%), however the hospital readmission rate was higher in this model (odds ratio = 1.57; 95%CI 1.02–2.44, p = 0.04; I2 = 90%).

**Conclusions:**

This study reveals a potential decrease in the length of stay for appendectomy patients using the DRG approach. However, no significant differences were observed in other outcomes analysis between the two approaches.

## Introduction

### Compensation models

Healthcare, like all activities, incurs costs. A major concern in the world is the rising cost of health care. World Health Organization data shows healthcare spending at 9.7% of the gross domestic product (GDP) of the planet, which is estimated around US$ 5.3 trillion. The same is observed in Brazil, where approximately R$ 74 billion are invested, 9% of the national GDP^1^. Here, we still have a perspective of increased expenses, that is proportional to the aging of the population, which should reach the same population profile of European countries such as Italy (22.6%)^2^. There is a positive correlation between the number of elderly people in the population and health expenses^3^. This relationship is demonstrably true and independent of the income level of the countries under study. In this persistent scenario, health expenditures are representative and may threaten the economic viability of health institutions. To counter this, efficient resource use, expense allocation, and healthcare payment system reforms are suggested^4^.

### Fee-for-service

The prevailing remuneration method in Brazilian health services is the fee-for-service (FFS), which falls under retrospective payment category. Traditionally used for medical and hospital services, FFS involves paying per procedure or item without controlling service quantity, potentially inflating bills. In this method, providers are compensated based on resources employed for each patient’s care^5^, contributing to expense increment and hospital costs inflation^6^.

The FFS approach may stimulate productivity by increasing the number, volume, and complexity of service. However, it does not consider the service quality, which should be the primary focus of healthcare delivery. In response to that, alternative models have emerged to address these new healthcare needs and prioritize care quality rather than mere volume^7^.

### Diagnosis related group payment

In essence, the DRG involves predetermined compensation for healthcare providers throughout the treatment of a specific condition. This amount covers all necessary patient care during treatment, making providers accountable for the entire treatment cycle. Thus, we can consider that this approach places the risk on the provider rather than sharing it with the payer^8^. The implementation of this remuneration model is expected to increase care and reduce the risks of complications and length of stay, ultimately leading to cost reduction and improved patient satisfaction. However, it’s worth noticing that the model’s success hinges on the payment package amount, which can introduce risks for the healthcare provider. Therefore, practical implementation needs a meticulous evaluation of provider risk strategies and payment amounts, as an inadequate value could pose significant financial risks for the provider^9^.

These questions have persisted since the inception of this model, necessitating ongoing studies to evaluate outcomes associated with new remuneration models or their adaptations. Therefore, the present work intended, through a comprehensive systematic review of the literature followed by a meta-analysis, to assess the actual effectiveness of the DRG model versus FFS, primarily focusing on appendectomy surgical procedure, a very prevalent condition, which leads to approximately US$ 3 billion in costs yearly^10^.

## Methods

### Kind of study

A systematic review of the literature and meta-analysis was carried out following the items of the Preferred Report Reporting Items for Systemic Reviews and Meta-Analyses (PRISMA)^11^. Table 1 shows the main variables of the present study that will be addressed according to the PECOS classification (P = Patients; E = exposition; C = Control; O = Outcomes; S = Study Design).

**Table 1:** PICOS table.

Patients	Patients submitted to appendectomy
Exposition	Budled Payment for Care Improvement payment model
Control	Fee-for-service payment model
Outcomes	Effectiveness of the payment model considering complications, costs and length of hospital stay
Study Design	Prospective Studies; Retrospectives (observational/epidemiological)

Source: Elaborated by the authors.

### Protocol registration

Following the choice of methodology chosen by the international platform for validating scientific papers EQUATOR, the order of materials and methods in this study was based on the PRISMA last updated listing. The present work was registered in the database International Prospective Register of Systematic Reviews (PROSPERO), from the Center for Reviews and Dissemination at the University of York (England), under the registration number CRD42023425872.

### Criteria in eligibility of the studies

We established as inclusion criteria observational studies published in the period from 2012 to 2022 whose clinical outcome was the evaluation of the comparison of remuneration for interventions and surgical procedures related to appendectomy in the traditional format, which is the fee per service or FFS to payment grouped by diagnosis called *bundled payment*, or variations in this remuneration system. We included studies which initially analyzed the effectiveness of these remuneration models in the appendectomy procedure only.

We excluded:

- Publications that did not correspond to the research theme;
- Publications of abstracts that did not contain the full text of the publications, preventing the complete interpretation of the data;
- Repeated publications;
- Those that approached the theme tangentially in relation to the objective of this study;
- Publications that addressed case reports or series of cases;
- Unfinished publications such as preprint and systematic or narrative reviews.

### Data sources and research strategy

We based the search strategy of this systematic revision considering the keywords “Bundled Payment for Care Improvement, Fee-For-Service, appendectomy, health care costs, diagnosis related group”. A search was carried out between March and May of 2022, and it was developed in databases of Scopus (Elsevier database and non-Elsevier data), PubMed (MEDLINE biomedical literature, life sciences journals and online books), and ScienceDirect (Elsevier database). We performed a combination of keywords with Boolean operators “OR” and “AND”. The same strategy was used in all databases.

We carried out the bibliographic search in three phases, and it was carried out by two evaluators. The search result was evaluated independently by the two authors of the work to increase the efficiency of the search. At the end of the search, disagreements were reviewed with a senior reviewer so that the decision on whether to include the article could be evaluated.

The first phase of surveying the articles consisted of analyzing the titles, an in this phase we were able to exclude articles that did not fit our research. In the second step, the abstracts of the articles were evaluated, and in a third step we analyzed the full texts to extract relevant data for our review and statistical synthesis.

Afterwards, two independent researchers extracted the data based on a predefined protocol, and again the disagreements were solved by a third one. These two authors independently extracted the data following predefined search criteria and quality assessment.

### Statistical analysis

Initially, we built a database in Excel, in which we transferred the relevant data that were collected in the included articles. These data were then analyzed in the statistical program Minitab 18 (version 18, Minitab, LLC, State College, Pennsylvania, United States of America). Forest plots were made in Review Manager (RevMan) Version 5.4 (The Cochrane Collaboration).

### Risk bias

Risk of bias was assessed in non-randomized studies with the risk of bias in non-randomized studies – of interventions tool (ROBINS-I)^12^. Two independent authors completed the risk of bias assessment. Disagreements were solved through a consensus after discussing reasons for discrepancy. 

## Results

### Selection of articles

**Figure 1:**
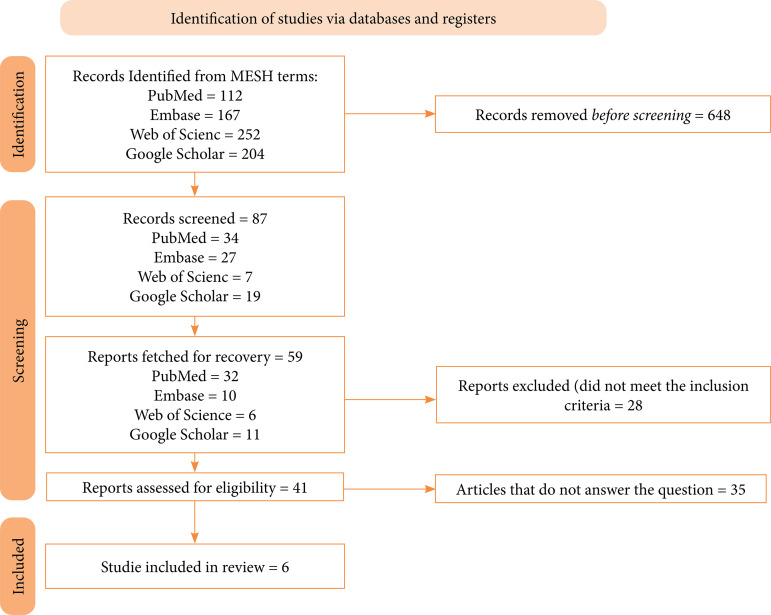
PRISMA diagram with the results found in the research.

Source: Elaborated by the authors.

In the initial phase of the search in all databases, 735 articles were identified. After an initial review, we identified 87 studies that were eligible. From this, we excluded 28 articles for not meeting the eligibility criteria previously defined and described in the methodology of our study. At the end, we included six articles in our work. The results found in the database search are described in the diagram of Fig. 1 13.

### Qualitative synthesis

The first study included was carried out by Kim et al.^14^, and the objective was to report the initial results considering clinical aspects and medical costs of the Korean DRG system for appendectomies. A prospective observational study with 416 patients was performed, and it evaluated clinical outcomes and medical costs associated with appendectomy before and after implementation of this model. It was demonstrated that the length of hospital stay was shorter after the implementation of DRG (2.98 ± 1.77 days vs. 3.82 ± 1.84 days; p < 0.001). No significant differences were observed in perioperative outcomes and medical costs, nor differences associated with follow-up costs after discharge, frequency of visits to the outpatient clinic or emergency department, or hospital readmissions. So, it was concluded that in the Korean DRG system for appendectomy, except for a shorter hospital stay, there were no significant differences in other outcomes.

The study by Kim et al.^15^, also conducted in Korea in 2015, evaluated the impact of the prospective payment system based on diagnostic groups on the use of medical resources and the rate of adverse events during laparoscopic appendectomy. Patients who underwent laparoscopic appendectomy between November 2012 and February 2014 were included, and the outcomes were also evaluated before and after the application of the model. After implementation of the DRG, the length of hospital stay decreased by 10% (4.9 days before vs. 4.4 days after DRG; p < 0.001). Initial hospital stay and total cost were significantly lower after establishing the DRG model (both p < 0.001). Complication rates during the initial hospital stay (3.5% before vs. 2.3% after DRG; p = 0.225) and readmission rates (4.3% vs. 2.5%, respectively; p = 0.227) were statistically similar. The authors concluded that implementation of the DRG for laparoscopic appendectomy had no negative effect on the rate of adverse events and reduced utilization of medical resources.

The third study evaluated the long-term result of the implementation of the DRG also in Korea and appendectomy procedures^16^. Clinical data of Korean patients who underwent appendectomy were retrospectively analyzed and divided in two groups: patients who received services before and after the implementation of the DRG system. Although the implementation of the DRG system for appendicitis significantly reduced the postoperative hospital stay (2.8 ± 1.0 days vs. 3.4 ± 1.9 days; p < 0.001), it did not reduce the total cost of hospitalization. The independent factors related to the total cost of hospitalization were patients aged 70 years old or older (odds ratio – OR 3.214; 95% confidence interval – 95%CI 1.769–5.840; p < 0.001) and surgical time greater than 100 minutes (OR 3.690; 95%CI 2.007–6.599; p < 0.001). Furthermore, older patients (? 70 years old) had 3.255 times greater relative risk of having a higher total in-hospital cost (95%CI 1.731–6.119; p < 0.001). Finally, it was concluded that the patient’s age should be considered as an important variable when evaluating the effectiveness of remuneration models.

It is worth saying that, since July 2013, the Korean government has imposed DRG implementation on all hospitals in the country. The objective of the fourth study included in our review was to assess the effects of mandatory DRG participation on various outcome metrics for appendectomy patients^17^. Data were collected from 280,062 patients who underwent appendectomy between 2007 and 2014. Mandatory implementation of the DRG payment system in South Korea has led to significant reductions in length of stay and readmission rates for these patients. However, we must consider that the study has a cross-sectional design, which limits the cause-and-effect association between the variables.

A work carried out by Zhang et al.^18^ was also included here. This trial corresponds to a retrospective study of 208 patients (from 20 hospitals) who underwent appendectomy. Data were obtained from databases of medical insurance information systems directly linked to hospital information systems. One hundred and thirty-three patients used the FFS system, and 75 used the fixed-fee-per-group system related to diagnosis. For those using the diagnosis-related group system, the mean length of stay (6.2 days) and the mean number of antimicrobials prescribed (2.4) per patient were significantly lower than for patients using the pay-as-you-go system by services (7.3 and 3 days, respectively; p = 0.018; p < 0.05). Also, there were no significant differences in post-surgical complications between the two systems. The diagnosis-related group system had lower medical costs for appendectomy compared to the FFS system, without sacrificing the quality of medical care.

The last article here included was the Suk-Bae Moon’s trial^19^, which was a retrospective study that counted with 60 pediatric patients who underwent appendectomy procedure. Thirty of them were the FFS group, and the other half was submitted to procedure after adoption of the DRG system. In this trial, a shorter mean hospital stay in DRG approach was observed, as well as no differences in readmission rates, and it was concluded that DRG system worked well in pediatric patients regarding cost-effectiveness and short-term hospital stay.

**Figure 2:**
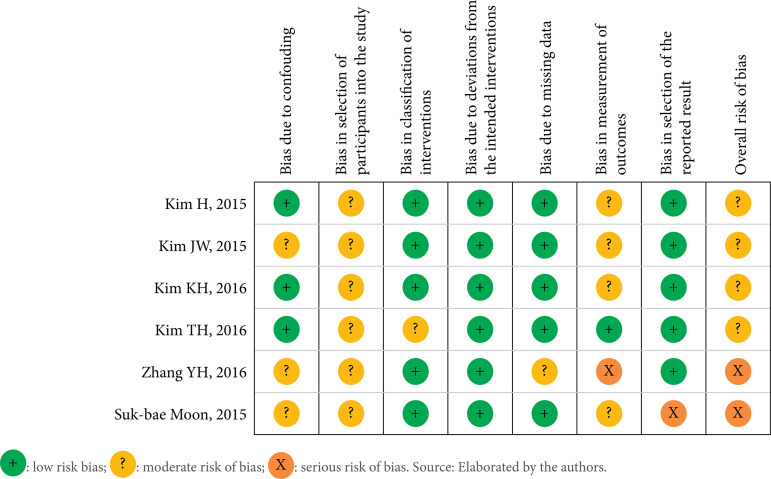
Risk of bias ROBINS-I of the included studies.

The articles exhibited an overall moderate bias, as assessed by the Robins-I score. Moreover, the studies conducted by Zhang et al.^18^ and Moon et al.^19^ demonstrated a higher degree of bias when compared to others, due bias in measurement of outcomes and in reported results, respectively (Fig. 2).

: low risk bias; : moderate risk of bias; : serious risk of bias. Source: Elaborated by the authors.

### Quantitative synthesis

#### Evaluation of complications

**Figure 3:**
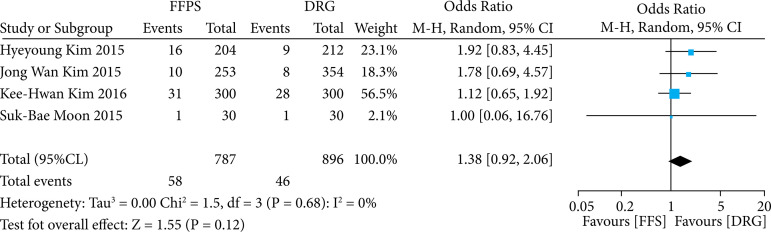
Complication rate as an outcome comparing health services remuneration models.

FFS: fee-for-service; DRG: diagnosis related group; 95% CI: 95% confidence interval. Source: Elaborated by the authors.

To evaluate the complication rate between the two remuneration models assessed in our study–FFS and DRG–, four observational studies were included. However, no statistically significant differences were observed (OR 1.38; 95%CI 0.92–2.06; p = 0.12; I^2^ = 0%), (Fig. 3).

#### Evaluation of costs associated with the intervention

**Figure 4:**
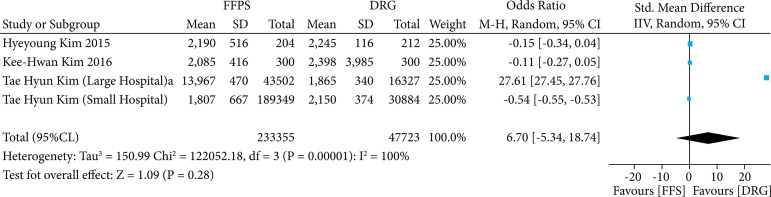
Comparison of remuneration models associated with appendectomy considering costs related to the procedure.

FFS: fee-for-service; DRG: diagnosis related group; 95% CI: 95% confidence interval; SD: standard deviation. Source: Elaborated by the authors.

Considering the costs associated with appendectomy, four observational studies were included in this analysis, and a random effect was applied due to heterogeneity. As we can see in the forest plot (Fig. 4), a single study showed a considerable result favoring the DRG model, however the result of the meta-analysis was not statistically significant (MD 6.70; 95%CI95 -5.34–18.74; p = 0.28; I^2^ = 100%).

#### Evaluation of length of hospital stay

**Figure 5:**
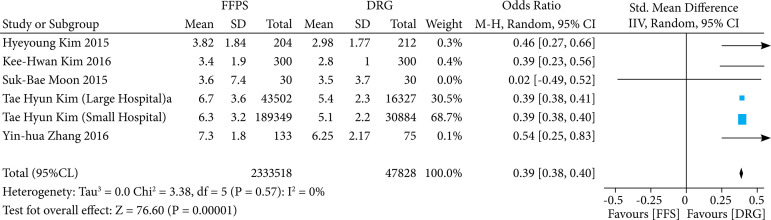
DRG versus FFS in terms of length of stay.

FFS: fee-for-service; DRG: diagnosis related group; 95% CI: 95% confidence interval; SD: standard deviation. Source: Elaborated by the authors.

When comparing the length of hospital stay between the two approaches, six studies made up our analysis (Fig. 5). Interestingly, this comparison showed statistically significance, once the length of stay was shorter in the DRG compensation model (MD 0.39; 95%CI 0.38–0.40; p < 0.00001; I^2^ = 0%).

#### Evaluation of hospital readmission and rate of outpatient visits

**Figure 6:**
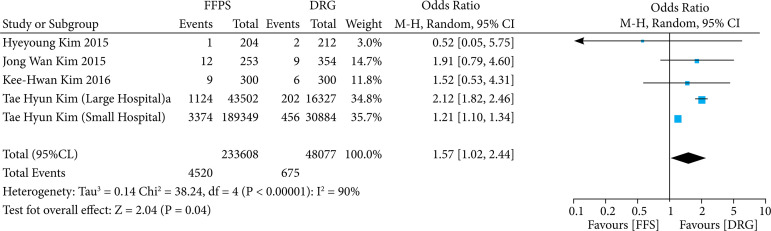
Evaluation of hospital readmission.

FFS: fee-for-service; DRG: diagnosis related group; 95% CI: 95% confidence interval. Source: Elaborated by the authors.

The analysis of hospital readmission rate in both remuneration models was performed with data extracted from five articles. The random effect was performed due to the heterogeneity rate of the studies, and a statistically significant difference was demonstrated (OR 1.57; 95%CI95 1.02–2.44; p = 0.04; I^2^ = 90%) (Fig. 6).

**Figure 7:**
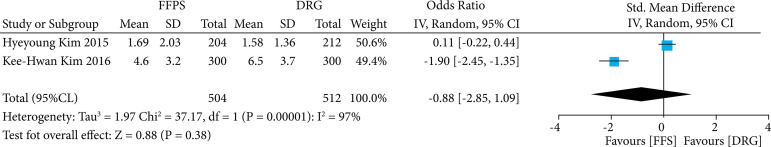
Evaluation of post-surgical outpatient care.

FFS: fee-for-service; DRG: diagnosis related group; 95% CI: 95% confidence interval; SD: standard deviation. Source: Elaborated by the authors.

Finally, considering the number of outpatient visits performed after appendectomy in the two remuneration models evaluated, two studies were included in this analysis. However, no significant difference was observed between the groups (MD -0.88; 95%CI -2.85–1.09; p = 0.38; I^2^ = 97%) (Fig. 7).

## Discussion

This systematic review assessed the fixed remuneration model’s effectiveness for appendectomy compared to the traditional pay-per-service model. Through a review of the medical literature, only appendectomy data was included. Our meta-analysis revealed no significant differences in final costs, complication rates, and post-surgery outpatient visits. However, the fixed payment model had a shorter hospital stay, but at a cost of a higher hospital readmission rate.

Literature lacks comprehensive systematic reviews and meta-analyses comparing health service remuneration models: fixed episode payments like bundled payment versus pay-per-service models. Existing studies mostly emphasize cost reduction due to shorter hospitalization times. However, research results on hospital readmission rates under the *bundled payment model* vary and sometimes conflict. Complication-related costs for acute appendicitis are higher, directly linked to longer hospital stays and increased likelihood of early readmission. The rise in early readmission rates suggests that the diagnosis-based fixed payment system might accelerate negative practices, such as premature discharge of patients still needing supervised recovery.

In the FFS, the traditional model, everything that is consumed in the provision of health services such as exams, procedures, materials, and hospitalizations will be added to an invoice that will be later sent to health plan operators (HPO) to proceed with the payment. In this case, the final amount to be received by the health team can vary greatly in the amount of remuneration, as this amount depends on the volume of services provided and materials consumed, which can lead the provider to perform more procedures and which will result in an increase in their remuneration, resulting in harmful waste of hospital bills^8^.

On the other hand, the *bundled payment* consists of ways of remunerating acts that are part of a treatment cycle, and this remuneration is not determined only by the cost of the intervention that was carried out, but rather by a global analysis of the possible consequences associated with this intervention. In this sense, the health institution responsible for providing the service is also responsible for complications that may occur during hospitalization and for immediate care in the post-hospital period and patient follow-up. A preliminary study assessing this fixed-payment approach indicated reduced costs for total joint arthroplasty. This reduction likely stems from shorter hospital stays, diminished readmission rates, and fewer referrals to rehabilitation units^9^.

While our meta-analysis of appendectomy studies did not showcase cost reduction under the fixed payment model, it did highlight a decrease in hospital stay duration linked to this model.

This outcome parallels earlier discoveries that the average hospital stays for patients with specific diagnoses decreased upon adopting the voluntary DRG system in South Korea between 2004 and 2011^20^. Comparable results surfaced in other nations that embraced the fixed payment model. A study in Germany evaluated this approach’s efficacy using dermatological procedures as a foundation^21^. Likewise, in Thailand, the model reduced hospitalization time following cardiac revascularization procedures^22^.

After these compensation approaches were first introduced as a pay system in the United States of America in 1983, several DRG-based systems were implemented worldwide. The primary aim of implementing these payment systems is usually the anticipation of heightened transparency in hospital performance and resource utilization through standardized reimbursement. This anticipation translates into improved efficiency in delivering appropriate care, while also discouraging unnecessary medical interventions^23^. In the creation of the DRG model, the principal figures involved in its inception regard it as a system for both cost and quality control concerning hospital performance. They recognize this model as a tool for enhancing the quality of hospital care, addressing waste as a key adversary to overcome. As aptly stated in the preface of the book *Diagnosis Related Group in Europe*, authored by Robert Fetter^24^, the mode “serves as a foundation for hospitals to evaluate their relative cost performance in comparison to other institutions and to formulate strategies for cost reduction”.

In this sense, new methods of healthcare remuneration are being developed and globally implemented to increase provider earnings while ensuring service quality. Recently, there’s been a push for performance-based remuneration models prioritizing patient care quality, transferring some risk and responsibility to providers. Besides the model reviewed here, other ones, like pay for performance, tie compensation to provider performance, assessing cost reduction and healthcare quality improvement. Doctor’s perspectives on cardiovascular disease management payments were studied, with initial data collected through interviews revealing concerns for care quality. Some favored economic incentives as recognition and motivation for better results. The study found potential for cost containment and improved care quality^25^.

In a Chinese literature review, prospective DRG-based payments were assessed for overall inpatient care effectiveness. While DRG payments reduced hospitalization time, other effects were inconclusive^26^. Like our study, hospitalization time decreased, but DRG showed higher readmission rates. The traditional FFS model is a wasteful and inefficient resource, unlike prospective payments, which aim to curb provider spending. While promising, the fixed payment model has limitations and may lead to cheaper, lower quality supplies and services. Patients with chronic conditions might face cost-based treatment choices. Patient selection and DRG categorization can lead to unexpected costs, impacting reimbursement^27^.

*Bundled* compensation model *payment* is important for patient discussion of atypical cases–the outliers, which are patients with diagnoses correctly placed in a certain DRG group and who, despite the efficiency in providing health care, will have complications during hospitalization resulting in an increased cost. Atypical cases were initially not fully anticipated in the DRG formulation. The United States of America has led to a voluntary roll-out of the bundled system and even the adoption of a hybrid form of FFS and one that offers rewards for spending reductions. Some countries calculate the outlier limit by adding two or three standard deviations to this average, as in Germany, Spain, and the United States Medicare. Other ones use a non-parametric estimate, adding 1.5 times the interquartile range to the third quartile, seen in England, Italy, and Denmark. France combines both methods for outlier determination^28^^,^ 29.

It should be noted that technological innovation is not encouraged within the prospective payments model. The allowances for the necessary errors inherent in technological exploration and advancement are constrained. The evident greater consumption resources in patient care, particularly within medical education and training institutions, is overlooked and even discouraged under this remuneration system. Also, there are no works available in the medical literature containing comparative data on the prospective remuneration system and the innovation and teaching of new physicians, once available articles comprise scholarly opinions and critiques within medical journal editorials. This raises the question of whether academic services necessitate or should consider an alternative form of remuneration and funding.

The limitations of the present study and its results are mainly associated with the quantity and quality of the studies included in the systematic review. The included studies were few, and most of them had a retrospective longitudinal design. There is no other possible design of primary studies that consider the theme. It is necessary to carry out more high-quality primary studies that assess the effect of this fixed-payment model, and new systematic reviews of these studies will be important for consolidating the findings and conclusions of the present work. In general, health costs increased in all analyzed scenarios–observing periods prior to the COVID-19 pandemic.

This framework leads to the imposition of challenges to managers regarding the economic viability of the system and maintains the growing interest in the discussion of alternative reimbursement payment models. The bundled model has the potential to significantly impact reimbursements to healthcare systems. Considering this scenario, forthcoming high-quality primary studies that examine the efficacy of the strategies employed emphasizing the analysis of replacing the traditional FFS payment model with an alternative model are crucial.

The new DRG models offer advantages, such as better cost variability control and enhanced healthcare coordination. A systematic review was conducted on this pay-for-performance model. Among the insights gleaned from this review, it was apparent that this form of remuneration effectively stimulates goal attainment within health systems, particularly for immediate and targeted actions necessitating minimal effort from healthcare providers^30^.

## Conclusion

Nevertheless, while a potential reduction in the length of stay for patients undergoing appendectomy through the DRG approach was evident, this study did not yield statistically significant distinctions in terms of costs, complication rates, and the count of outpatient visits linked to this compensation model, when compared to FFS. Further high-quality studies are needed to confirm our findings.

## Data Availability

The data will be available upon request.
